# Exercise training worsens cardiac performance in males but does not change ejection fraction and improves hypertrophy in females in a mouse model of metabolic syndrome

**DOI:** 10.1186/s13293-022-00414-6

**Published:** 2022-01-31

**Authors:** Melinda E. Tóth, Márta Sárközy, Gergő Szűcs, Brigitta Dukay, Petra Hajdu, Ágnes Zvara, László G. Puskás, Gábor J. Szebeni, Zsófia Ruppert, Csaba Csonka, Ferenc Kovács, András Kriston, Péter Horváth, Bence Kővári, Gábor Cserni, Tamás Csont, Miklós Sántha

**Affiliations:** 1 Laboratory of Animal Genetics and Molecular Neurobiology, Institute of Biochemistry, Biological Research Centre, Eötvös Loránd Research Network, Temesvári krt. 62, Szeged, 6726 Hungary; 2 MEDICS Research Group, Department of Biochemistry, University of Szeged Albert Szent-Györgyi Medical School, Dóm tér 9, Szeged, 6720 Hungary; 3 Interdisciplinary Center of Excellence, University of Szeged, Dugonics tér 13, Szeged, 6720 Hungary; 4 Laboratory of Functional Genomics, Biological Research Centre, Eötvös Loránd Research Network, Temesvári krt. 62, Szeged, 6726 Hungary; 5 Doctoral School in Biology, University of Szeged, Szeged, Hungary; 6 Synthetic and Systems Biology Unit, Institute of Biochemistry, Biological Research Centre, Eötvös Loránd Research Network, Temesvári krt. 62, Szeged, 6726 Hungary; 7 Single-Cell Technologies Ltd, Temesvári krt. 62, Szeged, 6726 Hungary; 8 Institute for Molecular Medicine Finland (FIMM), University of Helsinki, 00014 Helsinki, Finland; 9 Department of Pathology, Albert Szent-Györgyi Medical School, University of Szeged, Állomás utca 1, Szeged, 6720 Hungary

**Keywords:** Metabolic syndrome, Hyperlipidemia, Obesity, Endoplasmic reticulum stress, Sex-based differences, Endurance training, Cardio-metabolic disease, Heart failure, Left ventricular hypertrophy, Diastolic function

## Abstract

**Background:**

Metabolic syndrome (MetS) refers to a cluster of co-existing cardio-metabolic risk factors, including visceral obesity, dyslipidemia, hyperglycemia with insulin resistance, and hypertension. As there is a close link between MetS and cardiovascular diseases, we aimed to investigate the sex-based differences in MetS-associated heart failure (HF) and cardiovascular response to regular exercise training (ET).

**Methods:**

High-fat diet-fed male and female APOB-100 transgenic (HFD/APOB-100, 3 months) mice were used as MetS models, and age- and sex-matched C57BL/6 wild-type mice on standard diet served as healthy controls (SD/WT). Both the SD/WT and HFD/APOB-100 mice were divided into sedentary and ET groups, the latter running on a treadmill (0.9 km/h) for 45 min 5 times per week for 7 months. At month 9, transthoracic echocardiography was performed to monitor cardiac function and morphology. At the termination of the experiment at month 10, blood was collected for serum low-density lipoprotein (LDL)- and high-density lipoprotein (HDL)-cholesterol measurements and homeostatic assessment model for insulin resistance (HOMA-IR) calculation. Cardiomyocyte hypertrophy and fibrosis were assessed by histology. Left ventricular expressions of selected genes associated with metabolism, inflammation, and stress response were investigated by qPCR.

**Results:**

Both HFD/APOB-100 males and females developed obesity and hypercholesterolemia; however, only males showed insulin resistance. ET did not change these metabolic parameters. HFD/APOB-100 males showed echocardiographic signs of mild HF with dilated ventricles and thinner walls, whereas females presented the beginning of left ventricular hypertrophy. In response to ET, SD/WT males developed increased left ventricular volumes, whereas females responded with physiologic hypertrophy. Exercise-trained HFD/APOB-100 males presented worsening HF with reduced ejection fraction; however, ET did not change the ejection fraction and reversed the echocardiographic signs of left ventricular hypertrophy in HFD/APOB-100 females. The left ventricular expression of the leptin receptor was higher in females than males in the SD/WT groups. Left ventricular expression levels of stress response-related genes were higher in the exercise-trained HFD/APOB-100 males and exercise-trained SD/WT females than exercise-trained SD/WT males.

**Conclusions:**

HFD/APOB-100 mice showed sex-specific cardiovascular responses to MetS and ET; however, left ventricular gene expressions were similar between the groups except for leptin receptor and several stress response-related genes.

## Highlights

1. Both HFD/APOB-100 males and females developed obesity and hypercholesterolemia; however, only males presented insulin resistance. Exercise training did not change these metabolic parameters significantly.
2. HFD/APOB-100 males showed echocardiographic signs of mild heart failure with thinner walls and dilated ventricles, whereas females developed a starting left ventricular hypertrophy assessed by echocardiography and histology.
3. In response to exercise training, SD/WT males developed increased left ventricular volumes, and females presented physiologic hypertrophy.
4. Exercise-trained HFD/APOB-100 males presented worsening heart failure with reduced ejection fraction. On the contrary, exercise-trained HFD/APOB-100 females reversed the echocardiographic signs of left ventricular hypertrophy.
5. Sex, metabolic syndrome, and exercise training alter the gene expression pattern of the myocardium, which may be involved in the development of sex-specific cardiac alterations in the state of metabolic syndrome or to exercise training.

## Background

Cardio-metabolic diseases, including cardiovascular diseases (CVDs) and type 2 diabetes mellitus (T2DM), are the leading causes of morbidity and mortality worldwide [1]. However, cardio-metabolic diseases are strongly influenced by genetic factors [2]; they are predominantly caused by an unhealthy lifestyle, such as physical inactivity and excess calorie intake, which could lead to abdominal obesity [3, 4] and obesity-associated clustering of other cardio-metabolic risk factors, including impaired glucose regulation, dyslipidemia and hypertension [1, 5]. These cardio-metabolic risk factors occurring together are usually called metabolic syndrome (MetS) [2, 6]. The association of obesity and MetS with CVDs, including atrial fibrillation, ischemic heart disease, and heart failure (HF), is well recognized. In contrast, the deep mechanistic links between MetS and CVDs are not entirely clear yet [7]. Nevertheless, experimental studies have discovered some fundamental mechanisms contributing to the development of obesity and MetS and their direct links to CVDs [2, 7]. These mechanisms include, e.g., obesity-related inflammation, pro-thrombotic milieu, cell death mechanisms, mitochondrial dysfunction, increased nitro-oxidative and endoplasmic reticulum (ER) stress [2, 7].

We previously set up a high-fat diet-fed (7 months) APOB-100 transgenic mice model to induce MetS. In this MetS model, obesity was accompanied by elevated serum levels of triglyceride, fasting glucose, and TNFα, as well as hepatic steatosis, especially in male animals [8]. As the serum level of LDL and very-low-density lipoprotein (VLDL) is generally lower in rodents compared to humans, wild-type mice are more resistant to high-fat diet-induced cardio- and cerebrovascular abnormalities [9]. However, by overexpressing APOB-100, the serum lipid profile of the transgenic mice becomes similar to that of humans. Therefore, the high-fat diet-fed APOB-100 transgenic mouse strains are widely accepted and validated models of human hyperlipidemia and atherosclerosis [10–14]. However, the sex-based differences in the development of MetS and its cardiovascular complications, particularly MetS-associated HF, are not well characterized in this model yet.

In humans, several mechanisms and components of MetS show sex-specific differences [7, 15–17]. Although women have higher fat mass than men, the prevalence of MetS is lower in premenopausal women but higher in postmenopausal women than men at a similar age [7, 18]. It is also well-known that sex hormones directly regulate glucose and lipid metabolism. A decreased estrogen level or a relative increase in testosterone concentration could induce insulin resistance and a pro-atherogenic lipid profile [7, 18]. Indeed, ischemic heart disease, including acute myocardial infarction, is more common in men than in age-matched women [7, 18]. In contrast, cardio-metabolic HF caused by obesity, hypertension, T2DM, or MetS is more common in women. Interestingly, women more often develop the phenotype called heart failure with preserved ejection fraction (HFpEF) with left ventricular hypertrophy (LVH), whereas men more commonly present heart failure with reduced ejection fraction (HFrEF) with dilated ventricles and thinner walls [7, 16, 18].

Preventing cardiovascular complications in patients with MetS is of primary importance. However, pharmacologic treatments alone are not necessarily sufficient to achieve this goal. Using multimodality strategies, such as weight management and regular physical exercise beyond pharmacologic strategies, could be more effective [19, 20]. Increasing evidence suggests that regular exercise can restore metabolic abnormalities and prevent pre-mature cardiovascular mortality [21, 22]. In our previous study, fasting blood glucose level was significantly higher in HFD/APOB-100 males and females compared to sex-matched SD/WT mice, irrespective of ET [8]. Notably, HFD/APOB-100 males had significantly higher fasting blood glucose levels than HFD/APOB-100 females irrespective of ET, indicating more severe hyperglycemia in HFD/APO-B100 males [8]. However, there was no statistically significant difference in the serum insulin levels between the SD/WT and HFD/APOB-100 groups [8]. Several guidelines for HF treatment recommend exercise training (ET) as an alternative therapeutic modality; however, only limited data are available on the sex-based differences in the effectiveness of ET in HF patients [19, 23, 24]. Moreover, the effects and mechanisms of sex-based differences on ET in MetS-associated HF are not well-characterized yet. Therefore, in the present study, we aimed to investigate the sex-based differences in the development of MetS-associated cardiac abnormalities and cardiovascular response to regular ET in our previously setup MetS model.

## Methods

This investigation conformed to the EU Directive 2010/63/EU and was approved by the regional Animal Research Ethics Committee of Csongrád County (Csongrád county, Hungary; project license: XVI/766/2018). All institutional and national guidelines for the care and use of laboratory animals were followed.

### Animals

A total of 96 age-matched (3 months) male (*n* = 24 C57BL/6 wild type, 23–29 g, and *n* = 24 APOB-100, 21–30 g) and female (*n* = 24 C57BL/6 wild type, 19–25 g, and *n* = 24 APOB-100, 19–25 g) mice were used in this study. All mice were kept in the same room under controlled conditions (24 °C, 12–12 h light–dark cycle) throughout the experiment. Two to three mice were housed per cage, with food and water available ad libitum. The APOB-100 mouse strain overexpressing the human APOB-100 protein was previously established and used by our group [12, 25–27]. This APOB-100 mouse strain was bred and maintained in a hemizygous form on a C57BL/6 genetic background. Breeding of the transgenic mouse strain was approved by the regional Animal Research Ethics Committee (Csongrád county, Hungary; project license: XVI./2724/2017). To determine the genotype of hemizygous APOB-100 animals and wild-type littermates, DNA from tail biopsies of 10-day-old pups was purified, and the presence of the transgene was detected by PCR, using primers for the 5’ promoter region of the human APOB-100 gene [10].

### Experimental setup

**Fig. 1:**
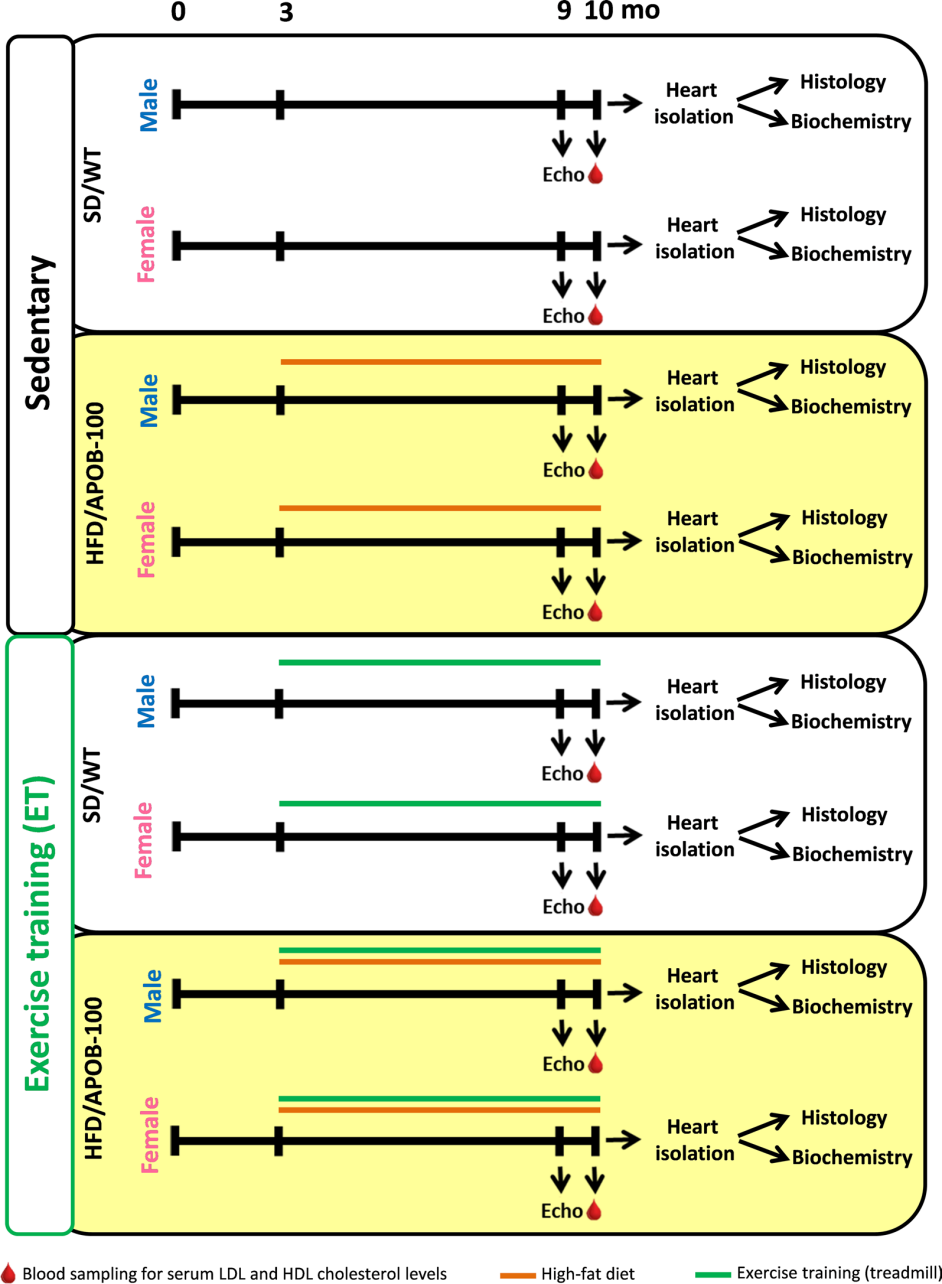
Protocol figure. Mice were divided into eight groups (*n* = 12/group). Male and female wild-type mice on a standard chow diet were used as healthy controls (SD/WT). Sex-matched APOB-100 mice were fed with a high-fat diet (to induce MetS and cardiovascular abnormalities (HFD/APOB-100). Both control and APOB-100 mice were divided into sedentary and exercise groups trained by treadmill running, five times a week, for 45 min per occasion, at a speed of 0.9 km/h. Dietary intervention and ET started at the age of 3 months and lasted for 7 months. At 9 months of age, transthoracic echocardiography was performed. At 10 months of age, all mice were terminally anesthetized by sodium pentobarbital, and fasting blood samples were collected through a cardiac puncture to measure serum low-density lipoprotein (LDL)-cholesterol, high-density lipoprotein (HDL)-cholesterol, blood glucose, and serum insulin levels. Before removing hearts, transcardial perfusion was performed. Weights of the hearts were measured, and then left and right ventricles were separated. Then a subgroup of left ventricular samples (*n* = 6/group) was frozen in liquid nitrogen for RNA isolation, and another subgroup of left ventricles (*n* = 6/group) was fixed in 4% paraformaldehyde

Animals were divided into eight groups, including 12 mice in each group as described in our previous study [8] (Fig. 1). Briefly, male and female wild-type mice on a standard chow diet were used as healthy controls (SD/WT) (Fig. 1). Sex-matched APOB-100 mice were fed with a high-fat diet (HFD, Special Diet Services, UK) to induce MetS and cardiovascular abnormalities (HFD/APOB-100) (Fig. 1) [12, 13]. For detailed diet composition, see our previous study using the same animals to characterize the molecular changes in the adipose tissue [8]. Both control and APOB-100 mice were divided into sedentary and exercise groups trained by treadmill running, five times a week, for 45 min per occasion, at a speed of 0.9 km/h (Fig. 1). Dietary intervention and ET started at the age of 3 months and lasted for 7 months (Fig. 1). At 9 months of age, transthoracic echocardiography was performed (Fig. 1). At 10 months of age, all mice were terminally anesthetized by sodium pentobarbital (*ip.* 150 µg/g). Then fasting blood samples were collected through a cardiac puncture to measure standard laboratory parameters, including serum low-density lipoprotein (LDL)-cholesterol, high-density lipoprotein (HDL)-cholesterol, blood glucose, and serum insulin levels. Before removing hearts, transcardial perfusion was performed (with 0.9% sodium chloride in 0.01 M phosphate-buffered saline [PBS], pH = 7.4). Weights of the hearts were measured, and then left and right ventricles were separated. Then a subgroup of left ventricular samples (*n* = 6/group) was frozen in liquid nitrogen for RNA isolation, and another subgroup of left ventricles (*n* = 6/group) was fixed in 4% paraformaldehyde (solved in 0.1 M PBS, pH = 7.4) for histology (Fig. 1).

### Transthoracic echocardiography

Cardiac morphology and function were assessed by transthoracic echocardiography at the age of 9 months, as we described previously [28]. Mice were anesthetized with 2% isoflurane (Forane, AESICA, Queenborough Limited Kent, UK). Then the chest was shaved, and the animal was placed supine on a heating pad. Two-dimensional, M-mode, Doppler, and tissue Doppler echocardiographic examinations were performed following the criteria of the American Society of Echocardiography with a Vivid IQ ultrasound system (General Electric Medical Systems, New York, NY, USA) using a linear array 4.5–13 MHz transducer (General Electric Medical Systems 12L-RS probe, New York, NY, USA) for morphology, and a phased array 5.0–11 MHz transducer (General Electric Medical Systems 12S-RS probe, New York, NY, USA) for function. Data of 3 consecutive heart cycles were analyzed (EchoPac Dimension software v201; General Electric Medical Systems New York, NY, USA) by an experienced investigator in a blinded manner. The mean values of three measurements were calculated and used for statistical evaluation. Systolic and diastolic wall thicknesses were obtained from parasternal short-axis view at the papillary muscles (anterior and inferior walls) and long-axis view at the level of the mitral valve (septal and posterior walls). The left ventricular diameters were measured using M-mode echocardiography from short-axis and long-axis views between the endocardial borders. Fractional shortening (FS) was used as a measure of cardiac contractility (FS = (left ventricular end-diastolic diameter [LVEDD] − left ventricular end-systolic diameter [LVESD])/LVEDD × 100). Ejection fraction was assessed by the Teichholz method on parasternal short-axis M-mode images and used as a measure of global systolic function. Further functional parameters, including left ventricular end-diastolic volume (LVEDV) and left ventricular end-systolic volume (LVESV), were calculated on four-chamber view images delineating the endocardial borders in diastole and systole. The stroke volume was calculated as the difference between LVEDV and LVESV. Cardiac output was calculated as the product of the stroke volume and heart rate. Diastolic function was assessed using pulse-wave Doppler across the mitral valve and tissue Doppler of the mitral annulus from the apical four-chamber view. Isovolumic relaxation time (IVRT), early (E) and atrial (A) flow velocities, and septal mitral annular velocity (e’), as well as E/A and E/e’ ratios, provided an assessment of diastolic function. Heart rate was calculated using pulse-wave Doppler images during the measurement of transvalvular flow velocity profiles according to the length of 3 consecutive heart cycles measured between the start points of the E-waves.

### Serum LDL-cholesterol and HDL-cholesterol levels

To investigate the effects of sex and ET on the development of dyslipidemia as a diagnostic criterion of MetS, serum LDL-cholesterol and HDL-cholesterol levels were measured as described previously [29]. Blood samples, collected by cardiac puncture, were centrifuged at 4 °C, 1000×*g* for 10 min. Finally, the serum was separated and stored at –80 °C until use. The serum LDL- and HDL-cholesterol levels were measured in triplicate, carried out using commercially available enzymatic colorimetric assay kits according to the manufacturer’s instructions (Diagnosticum Ltd., Budapest, Hungary). Accuracy was tested using Standard Lipid Controls (Diagnosticum Ltd., Budapest, Hungary). Serum HDL-cholesterol and LDL-cholesterol concentrations were determined by measuring the absorbance of the blue-colored product at 596 nm with a microplate reader (FLUOstar OPTIMA, BMG Labtech, Ortenberg, Germany). Values were expressed in mmol/Liter.

### HOMA-IR index

Fasting serum glucose and insulin levels were measured to investigate the effects of sex and ET on the development of hyperglycemia and hyperinsulinemia as characteristic features of MetS, as described previously [8]. Briefly, mice were fasted overnight (12 h) before serum glucose level measurements. Blood samples were collected by cardiac puncture at the termination of the experiment (Fig. 1). Blood glucose levels were measured using AccuCheck blood glucose monitoring systems (Roche Diagnostics Corporation, Indianapolis, USA) as described previously [8, 28]. Serum insulin level and three other protein concentrations (leptin, resistin, and TNFα) were measured by immunoassay (MILLIPLEX® Mouse Adipokine Multiplex Immunoassay, MADKMAG-71 K, Merck Millipore, Darmstadt, Germany) according to the instructions of the manufacturer as described in our previous study using the same animals [8]. The serum levels of insulin, leptin, resistin, and TNFα are published in our previous article [8]. The widely used homeostatic model assessment for insulin resistance (HOMA-IR) index [30, 31] was calculated to estimate insulin resistance in APOB-100 mice by multiplying fasting serum insulin (μg/mL) with fasting blood glucose (mmol/L), then dividing by the constant 22.5 (i.e., HOMA-IR = [fasting serum insulin concentration × fasting blood glucose concentration]/22.5).

### Histological measurements on hematoxylin–eosin and picrosirius red/fast green stained sections

In a subgroup of the animals (*n* = 6 in each group), 5 μm thick transverse cut sections of the formalin-fixed paraffin-embedded subvalvular areas of the left ventricles were stained with hematoxylin–eosin (HE) or picrosirius red and fast green (PSFG) as described previously [32]. Histological slides were scanned with a Pannoramic Midi II scanner (3D-Histech, Budapest, Hungary), and digital images at the magnification of ×10, ×40, and ×100 were captured. On the digital HE images, cardiomyocyte cross-sectional areas were measured to investigate the effects of sex, MetS, and ET on cardiac morphology at the cellular level. The Biology Image Analysis Software (BIAS, Single-Cell Technologies Ltd., Szeged, Hungary) was used for the evaluation of HE-stained slides [32]. Image pre-processing was followed by deep learning-based cytoplasm segmentation. User-selected objects were forwarded to the feature extraction module, configurable to extract properties from the selected cell components. Cardiomyocyte cross-sectional areas were measured by the software in 100 selected, longitudinally oriented, mono-nucleated cardiomyocytes on digital images from a single left ventricular transverse slide. Cardiac fibrosis was assessed on PSFG slides with an in-house developed program described previously [32]. Briefly, this program determines the proportion of red pixels of heart sections using two simple color filters. For each Red–Green–Blue (RGB) pixel, the program calculates the color of the pixel in Hue–Saturation–Luminance (HSL) color space. The first filter is used for detecting red portions of the image. The second filter excludes any white (empty) or light grey (residual dirt on the slide) pixel from further processing using a simple RGB threshold. In this way, the program groups each pixel into one of two sets: pixels considered red and pixels considered green but neither white nor grey. Red pixels in the first set represent collagen content and fibrosis. Green pixels in the second set correspond to cardiac muscle. The mean values of 10 representative images were calculated and used for statistical evaluation in the case of each left ventricular slide. Medium-size vessels and their perivascular connective tissue sheet, the subepicardial and subendocardial areas were avoided as much as possible.

### Quantitative real-time polymerase chain reaction (qPCR)

In another subgroup of the animals (*n* = 6 in each group), total RNA was isolated from left ventricular samples using an RNeasy Fibrous Tissue Mini Kit (Qiagen, Hilden, Germany) according to the manufacturer’s instructions. High Capacity cDNA Reverse Transcription Kit (Thermo Fisher Scientific, Waltham, Massachusetts, USA) was used to convert RNA samples to cDNA. Each reaction mixture contained 1 µg RNA (15 µL), 1.5 µL MultiScribe Reverse Transcriptase, 3 µL primer, 1.2 µL dNTP, 3 µL buffer, 6.3 µL RNase-free water. Parameters for the reverse transcription program were the following: incubation at 25 °C for 10 min, reverse transcription at 37 °C for 2 h, and inactivation at 85 °C for 5 min (using BioRad T100 Thermal Cycler, Hercules, CA, USA). The cDNA product was finally diluted at 1:20 and used as a qPCR reaction template. For the qPCR reaction, 10 µL cDNA, 1 µL (250 nM final) primer mix (forward + reverse), and 10 µL Power SYBR Green PCR Master Mix 2x (Thermo Fisher Scientific, Waltham, Massachusetts, USA) were mixed. Each reaction was performed in a total volume of 20 µL, and was run on a RotorGene 3000 instrument (Qiagen, Hilden, Germany) with the following settings: heat activation at 95 °C for 10 min; followed by 40 cycles of denaturation at 95 °C for 15 s, annealing at 60 °C for 60 s as described previously [8]. Melting curve analysis was performed between 50 and 95 °C to verify the specificity of the amplification. Primer sequences used in qPCR reactions are listed in Table 1. The mouse *Gapdh* gene served as an internal control for normalization. Relative gene expression levels were calculated using the ^ΔΔ^Ct method.

**Table 1:** Primer sequences

Protein name	Gene symbol	Forward primer	Reverse primer
Leptin receptor	*Lepr*	AGCTAGGTGTAAACTGGGACA	GCAGAGGCGAATCATCTATGAC
Adiponectin receptor protein 1	*AdipoR1*	TGGTCTTCGGGATGTTCTTC	CCCTGAATAGTCCAGTTTGGAA
Platelet glycoprotein 4	*Cd36*	TTGAAAAGTCTCGGACATTGAG	TCAGATCCGAACACAGCGTA
Solute carrier family 2, facilitated glucose transporter member 4	*Slc2a4*	ACACTGGTCCTAGCTGTATTCT	CCAGCCACGTTGCATTGTA
Insulin receptor	*Insr*	TCAAGACCAGACCCGAAGATT	TCTCGAAGATAACCAGGGCATAG
Insulin receptor substrate 1	*Irs1*	CGATGGCTTCTCAGACGTG	CAGCCCGCTTGTTGATGTTG
Tumor necrosis factor	*Tnf*	CCCTCACACTCAGATCATCTTCT	GCTACGACGTGGGCTACAG
Interleukin-1 beta	*Il1b*	GCAACTGTTCCTGAACTCAACT	ATCTTTTGGGGTCCGTCAACT
Transforming growth factor beta-1	*Tgfb1*	CTCCCGTGGCTTCTAGTGC	GCCTTAGTTTGGACAGGATCTG
Interleukin-10	*Il10*	CAGAGCCACATGCTCCTAGA	TGTCCAGCTGGTCCTTTGTT
Heat shock factor protein 1	*Hsf1*	GGGAAACAGGAGTGTATGGACT	CTTGTTGACAACTTTTTGCTGCT
Heat shock protein beta-1	*HspB1*	ATCCCCTGAGGGCACACTTA	GGAATGGTGATCTCCGCTGAC
Alpha(B)-crystallin	*HspB5*	GTTCTTCGGAGAGCACCTGTT	GAGAGTCCGGTGTCAATCCAG
Heat shock protein beta-3	*HspB3*	AGACCCCAGTGCGTTATCAG	GCAGTGCGTATAGTGTATGATCC
Heat shock protein beta-6	*HspB6*	TGTCCACGGACTCTGGGTATT	CACATGGTCGTCAACCACCTT
Heat shock protein beta-8	*HspB8*	AGACCCCTTTCGGGACTCA	GGCTGTCAAGTCGTCTGGAA
Heat shock protein 40	*Hsp40*	TTCGACCGCTATGGAGAGGAA	CACCGAAGAACTCAGCAAACA
Heat shock protein 70	*Hsp70*	GAGATCGACTCTCTGTTCGAGG	GCCCGTTGAAGAAGTCCTG
Carboxyl-terminus of Hsp70 Interacting Protein	*Chip*	CGGCAGCCCTGATAAGAGC	CACAAGTGGGTTCCGAGTGAT
Bcl-2-associated athanogene 1	*Bag-1*	GCAGCAGGGAGTTGACTAGAA	TTACTTCCTCGGTTTGGGTCG
Glucose-regulated protein 94	*Grp94*	AAGAATGAAGGAAAAACAGGACAAAA	CAAATGGAGAAGATTCCGCC
C/EBP homologous protein	*Chop*	CCACCACACCTGAAAGCAGAA	AGGTGAAAGGCAGGGACTCA
Binding immunoglobulin protein	*Bip*	TTCAGCCAATTATCAGCAAACTCT	TTTTCTGATGTATCCTCTTCACCAGT
X-box-binding protein 1 total	*Xbp1 total*	TGGCCGGGTCTGCTGAGTCCG	GTCCATGGGAAGATGTTCTGG
X-box-binding protein 1 unspliced	*Xbp1us*	CAGCACTCAGACTATGTGCA	GTCCATGGGAAGATGTTCTGG
X-box-binding protein 1 spliced	*Xbp1s*	CTGAGTCCGAATCAGGTGCAG	GTCCATGGGAAGATGTTCTGG
Glyceraldehyde-3-phosphate dehydrogenase	*Gapdh*	GGGTTCCTATAAATACGGACTGC	CCATTTTGTCTACGGGACGA

List of primer sequences used in qPCR study to evaluate expression levels of genes involved in the regulation of metabolism, inflammation, and stress response/ER stress. Names of proteins encoded by the genes are also listed

### Statistical analysis

Statistical analysis was performed using Sigmaplot 12.0 for Windows (Systat Software Inc., San Jose, CA, USA). The level of statistical significance was set at *p* < 0.05. All values are presented as mean ± SEM. The corresponding table or figure legend describes specific sample numbers used for measurements. The normal distribution of the data was checked by the Shapiro–Wilk normality test. Serum, morphometric, histologic, and several echocardiographic data showed normal distribution. In these cases, Three-Way Analysis of Variance (ANOVA) was performed first. In the case of statistical significance between the groups, the Holm–Sidak post hoc test was used. Then, to better characterize which sub-groups differ from the others significantly, data were further analyzed using two-way ANOVA followed by Holm–Sidak post hoc test within the female and male groups separately. Due to the opposite changes in several groups, the effect of sex was also tested by Student’s *t* test (in case of normal distribution) or Mann–Whitney U-test (if normality test failed) in the case of echocardiographic data as indicated in the Results and legends of Fig. 4 and Table 2. In the cases of HOMA-IR and several echocardiographic parameters, including systolic inferior wall thickness and E-velocity within males as well as diastolic septal, anterior, and inferior wall thicknesses, left ventricular end-systolic diameter, fractional shortening, e’ velocity and E/A within females, the normality test failed. Therefore, the Kruskal–Wallis test by ranks (i.e., ANOVA on ranks) was performed. In case of significant difference between the groups, the Holm-Sidak post hoc test was used after ANOVA on ranks. qPCR data are presented as % of the corresponding control group. In the case of gene expressional changes, normal distribution was checked using the Shapiro–Wilk normality test. In the case of normal distribution, the parametric Student’s t-test was performed for pairwise comparisons. If the normal distribution test failed, the non-parametric Mann–Whitney U-test was used for pairwise comparisons. (It is indicated in the Results, corresponding tables, and figure legends which statistical and post hoc tests were used in case of specific parameters).

**Table 2:** Echocardiographic parameters

Parameter (unit)	Sedentary				ET			
	SD/WT		HFD/APOB-100		SD/WT		HFD/APOB-100	
	Male	Female	Male	Female	Male	Female	Male	Female
PWTs (mm)	1.59 ± 0.09	1.32 ± 0.10	1.45 ± 0.10	1.48 ± 0.07	1.61 ± 0.13	1.69 ± 0.11^#^	1.46 ± 0.09	1.27 ± 0.07*
PWTd (mm)	1.33 ± 0.07	1.17 ± 0.17	1.15 ± 0.10	1.29 ± 0.06	1.4 ± 0.12	1.41 ± 0.11	1.23 ± 0.07	0.97 ± 0.05*
SWTs (mm)	1.57 ± 0.09	1.57 ± 0.13	1.37 ± 0.06	1.51 ± 0.07	1.59 ± 0.07	1.67 ± 0.09	1.49 ± 0.10	1.35 ± 0.03^$^*
SWTd (mm)	1.12 ± 0.09	1.27 ± 0.12	0.97 ± 0.03	1.13 ± 0.08	1.06 ± 0.05	1.21 ± 0.09	1.09 ± 0.07	0.94 ± 0.03*
AWTs (mm)	1.49 ± 0.06	1.54 ± 0.06	1.42 ± 0.04	1.65 ± 0.06^$^	1.48 ± 0.06	1.54 ± 0.08	1.27 ± 0.05*	1.3 ± 0.05*^#^
AWTd (mm)	1.05 ± 0.06	0.99 ± 0.07	1.02 ± 0.04	1.2 ± 0.04^$^*	1 ± 0.04	1.12 ± 0.10*	0.96 ± 0.04	0.9 ± 0.03*
IWTs (mm)	1.49 ± 0.10	1.24 ± 0.06^$^	1.32 ± 0.10	1.3 ± 0.09	1.6 ± 0.07	1.47 ± 0.10	1.29 ± 0.09*	1.4 ± 0.08
IWTd (mm)	1.21 ± 0.06	0.96 ± 0.08^$^	1.12 ± 0.09	1.1 ± 0.05	1.18 ± 0.06	1.26 ± 0.1^#^	1.09 ± 0.07	1.13 ± 0.07
LVEDD (mm)—long	3.66 ± 0.18	3.5 ± 0.21	4.04 ± 0.15	3.22 ± 0.16^$^	3.56 ± 0.16	3.2 ± 0.15	3.74 ± 0.11	3.42 ± 0.23
LVESD (mm)—long	2.33 ± 0.12	2.33 ± 0.25	2.76 ± 0.14^*^	2.04 ± 0.17^$^	2.03 ± 0.14	2.05 ± 0.19	2.65 ± 0.13^*^	2.51 ± 0.14
LVEDD (mm)—cross	3.92 ± 0.13	4.04 ± 0.19	3.8 ± 0.18	3.22 ± 0.10^$^*	3.72 ± 0.18	3.17 ± 0.13^$#^	3.51 ± 0.11	3.13 ± 0.16
LVESD (mm)—cross	2.59 ± 0.16	2.53 ± 0.21	2.57 ± 0.17	1.98 ± 0.10^$^*	2.09 ± 0.17	1.86 ± 0.11^#^	2.55 ± 0.11*	2.1 ± 0.16^$^
LVEDV (μL)	10.15 ± 0.72	6.92 ± 0.62^$^	8.17 ± 0.84	9.15 ± 0.86	13.09 ± 1.22^#^	9.2 ± 1.6	11.45 ± 1.10^#^	10.14 ± 1.23
LVESV (μL)	3.87 ± 0.27	3.11 ± 0.33	3.32 ± 0.56	3.68 ± 0.39	4.75 ± 0.44	4.38 ± 0.86	4.55 ± 0.43^#^	3.95 ± 0.32
MV E/A	2.3 ± 0.17	2.26 ± 0.41	2.13 ± 0.27	1.7 ± 0.28	1.7 ± 0.14	2.26 ± 0.27	1.9 ± 0.25	2.26 ± 0.29
MV E/e' septal	18.3 ± 1.1	15.3 ± 1.2	18.3 ± 1.6	13.9 ± 1.3^$^	16.1 ± 0.9	16.4 ± 0.9	17.2 ± 1.0	18.5 ± 1.1*^#^
IVRT (ms)	16.3 ± 1.3	17.7 ± 1.7	15.8 ± 0.1	15.9 ± 2.0	18 ± 0.8	16.5 ± 1.3	14.2 ± 0.9*	14.5 ± 0.6

AWT, anterior wall thickness; CO, cardiac output; cross, cross-sectional; d, diastolic; E-wave, early ventricular filling velocity; e', e'-wave; long, longitudinal, mitral annulus velocity measured by tissue Doppler; IVRT, isovolumic relaxation time; IWT, inferior wall thickness; LVEDD, left ventricular end-diastolic diameter; LVEDV, left ventricular end-diastolic volume; LVESD, left ventricular end-systolic diameter; LVESV, left ventricular end-systolic volume; MV, mitral valve; PWT, posterior wall thickness; s, systolic; SWT, septal wall thickness

Transthoracic echocardiographic was performed at 9 months of age in sedentary and exercise-trained (ET) male and female standard diet-fed wild-type (SD/WT) and high-fat diet-fed APOB-100 transgenic (HFD/APOB-100) mice. Values are mean ± SEM, *n* = 11–12/group, ^$^*p* < 0.05, female vs. male groups, *p < 0.05, HFD/APOB-100 vs. SD/WT groups, ^#^*p* < 0.05, ET vs. sedentary groups; Two-Way ANOVA (Holm-Sidak post hoc test) for comparisons between HFD/APOB-100 vs. SD/WT groups and ET vs. sedentary groups, and Student’s t-test or Mann–Whitney-U test for comparison between male and female groups. Kruskal–Wallis test followed by Holm-Sidak post hoc test was used in the cases of systolic inferior wall thickness within the male groups and diastolic septal, anterior, and inferior wall thicknesses, left ventricular end-systolic diameter, and E/A parameter within the female groups

## Results

### Exercise training did not improve the serum LDL-cholesterol and HDL-cholesterol levels in male and female HFD/APOB-100 mice

**Fig. 2:**
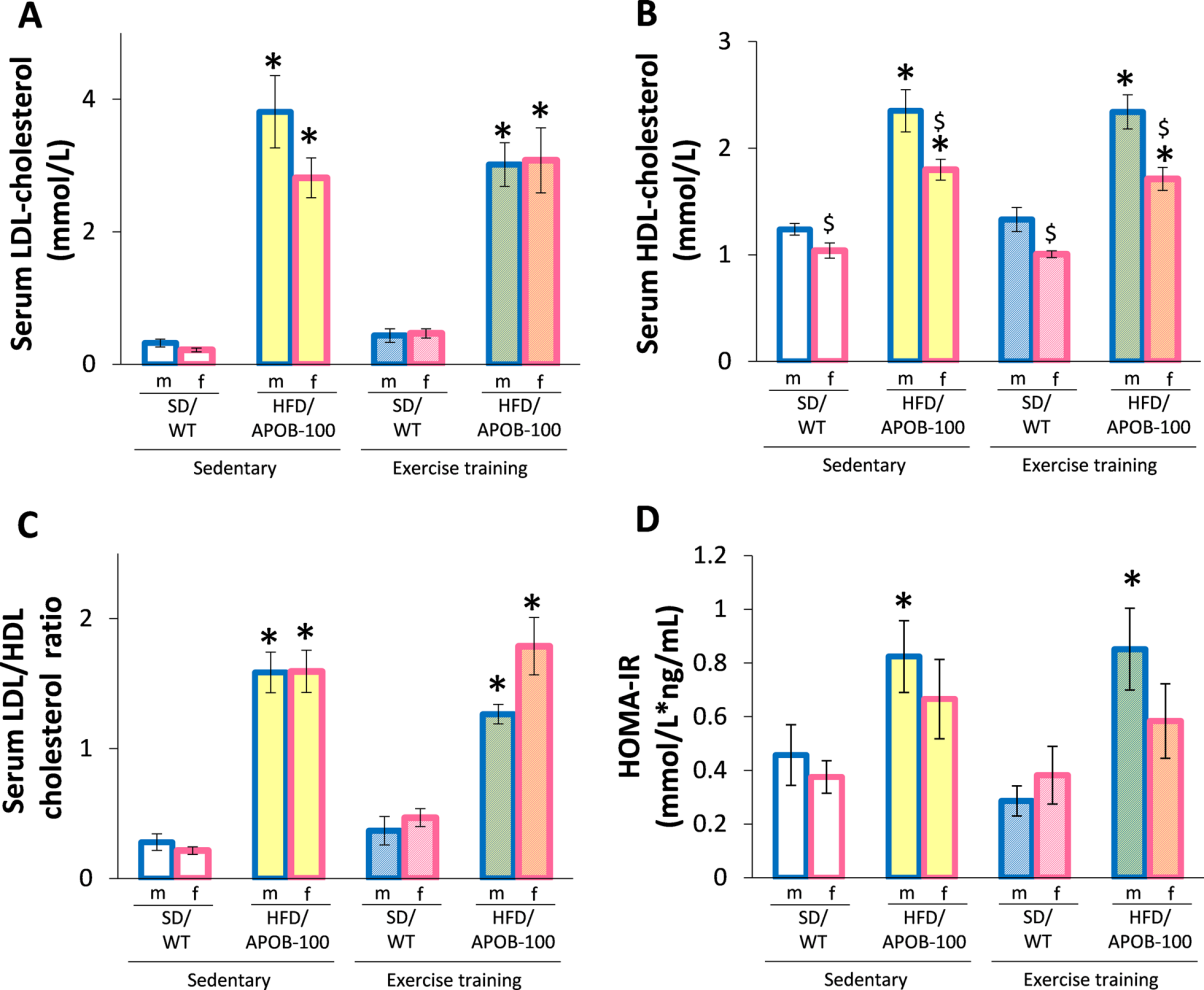
The effects of sex, MetS, and exercise training on serum laboratory parameters at 10 months of age in sedentary and exercise-trained male (m) and female (f) standard diet-fed wild-type (SD/WT) and high-fat diet-fed APOB-100 transgenic (HFD/APOB-100) mice. **A** Serum LDL-cholesterol level, **B** Serum HDL-cholesterol level, **C** Serum LDL/HDL-cholesterol ratio, and **D** HOMA-IR. Values are mean ± SEM, *n* = 11–12/group, ^$^*p* < 0.05, female vs. male groups, *p < 0.05, HFD/APOB-100 vs. SD/WT groups, Three-Way ANOVA (Holm-Sidak post hoc test) in cases of serum LDL-cholesterol, serum HDL-cholesterol and serum LDL/HDL cholesterol ratio. In the case of HOMA-IR values, Kruskal–Wallis test (ANOVA on ranks) was used followed by Holm-Sidak post hoc test

To investigate the effects of sex and ET on the development of hypercholesterolemia and insulin resistance in the HFD/APOB-100 mice, serum LDL-cholesterol and HDL-cholesterol levels and their ratio were measured as well as the HOMA-IR was calculated using the following formula: (fasting serum insulin concentration × fasting blood glucose concentration)/22.5 (Fig. 2A–D). The serum LDL concentration was significantly increased in the HFD/APOB-100 males and females compared to the sex-matched SD/WT controls irrespective of ET (*p < 0.05, Fig. 2A). There was no significant difference in the serum LDL-cholesterol level in SD/WT mice between sedentary and exercise-trained groups in both sexes (Fig. 2A). The serum HDL-cholesterol level was significantly increased in the HFD/APOB-100 males and females compared to the sex-matched SD/WT controls irrespective of ET proving the development of hypercholesterolemia in the HFD/APOB-100 mice (*p < 0.05, Fig. 2B). Interestingly, females had significantly lower serum HDL levels in both the SD/WT and HFD/APOB-100 mice irrespective of ET compared to group-matched males (^$^*p* < 0.05, Fig. 2B). To further investigate the effects of sex and exercise on cardiovascular risk, the ratio of serum LDL-cholesterol and HDL-cholesterol levels were measured (Fig. 2C). The serum LDL-cholesterol to HDL-cholesterol ratio was markedly increased in the HFD/APOB-100 males and females compared to the sex-matched SD/WT controls irrespective of ET (*p < 0.05, Fig. 2C). Interestingly, only male HFD/APOB-100 mice had significantly higher HOMA-IR values compared to the sex-matched SD/WT mice, irrespective of ET, pointing out the development of more severe insulin resistance in males (*p < 0.05, Fig. 2D). However, it should be mentioned that sedentary female HFD/APOB-100 mice had a tendency to increase in HOMA-IR value (*p* = 0.092) compared to the sedentary female SD/WT control group (Fig. 2D). In response to exercise, there was no tendency of increase in the HOMA-IR between female HFD/APOB-100 and sex-matched SD/WT mice, suggesting that HFD/APOB-100 females can benefit more from ET than HFD/APOB-100 males (Fig. 2D).

### Exercise training reduced the body weight in HFD/APOB-100 mice

**Fig. 3:**
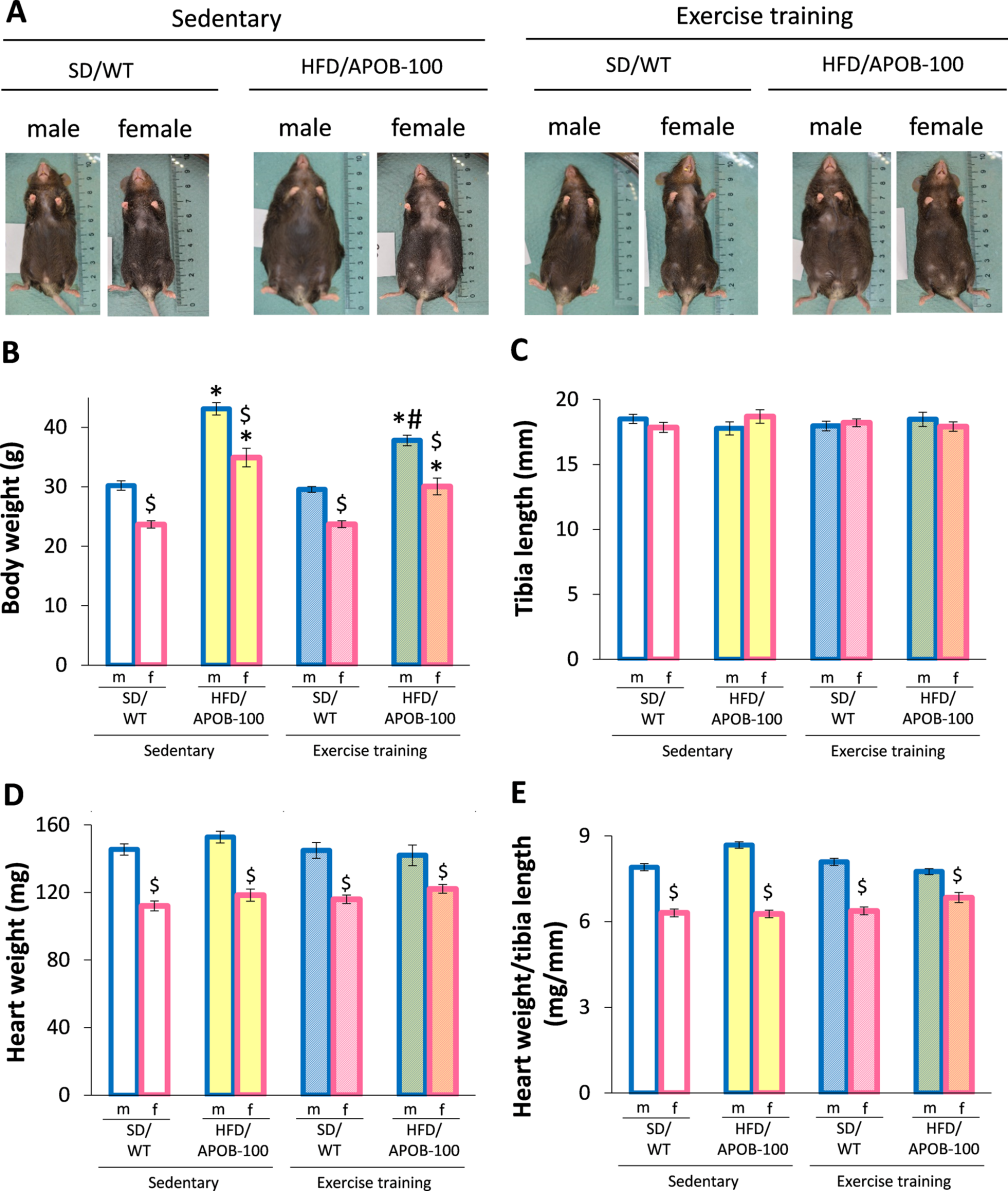
The effects of sex, MetS, and exercise training on body weight, heart weight and tibia length at 10 months of age in sedentary and exercise-trained male (m) and female (f) standard diet-fed wild-type (SD/WT) and high-fat diet-fed APOB-100 transgenic (HFD/APOB-100) mice. **A** Representative images of the mice in each group, **B** Body weight, **C** Tibia length, **D** Heart weight, and **E** Heart weight to tibia length ratio. Values are mean ± SEM, *n* = 11–12/group, ^$^*p* < 0.05, female vs. male groups, *p < 0.05, HFD/APOB-100 vs. SD/WT groups, ^#^*p* < 0.05, ET vs. sedentary groups; Three-Way ANOVA (Holm-Sidak post hoc test)

Morphological parameters including body weight, tibia length, and heart weight were measured at the termination of the experiment to investigate whether MetS or exercise influences ex vivo parameters similarly in both sexes (Fig. 3A–E). Irrespective of genotype or ET, the body weight was significantly smaller in females compared to the group-matched males (^$^*p* < 0.05, Fig. 3A, B) [8]. Both HFD/APOB-100 males and females had significantly increased body weight compared to the sex-matched SD/WT controls (*p < 0.05, Fig. 3B), irrespective of ET [8]. In response to ET, only male HFD/APOB-100 mice had significantly reduced body weight compared to the sedentary male HFD/APOB-100 mice (^#^*p* < 0.05, Fig. 3B) [8]. It should also be mentioned that ET reduced the body weight in female HFD/APOB-100 mice compared to the sex-matched sedentary HFD/APOB-100group using Student’s t-test for pairwise comparison (*p* = ^#^0.03). There was no significant difference in the tibia length between the groups (Fig. 3C). Similarly to the body weight, heart weight was significantly lower in females compared to the group-matched males irrespective of genotype or ET (^$^*p* < 0.05), indicating the significantly smaller body and heart size of females (Fig. 3D). Indeed, the heart weight to tibia length ratio was also significantly lower in females compared to the group-matched males irrespective of genotype or ET (^$^*p* < 0.05, Fig. 3E).

### Male and female hearts responded differently to metabolic syndrome and exercise training based on echocardiography

#### Sex-based differences in the echocardiogrpahic morphologic and functional parameters in the sedentary SD/WD groups due to the smaller heart size of females

**Fig. 4:**
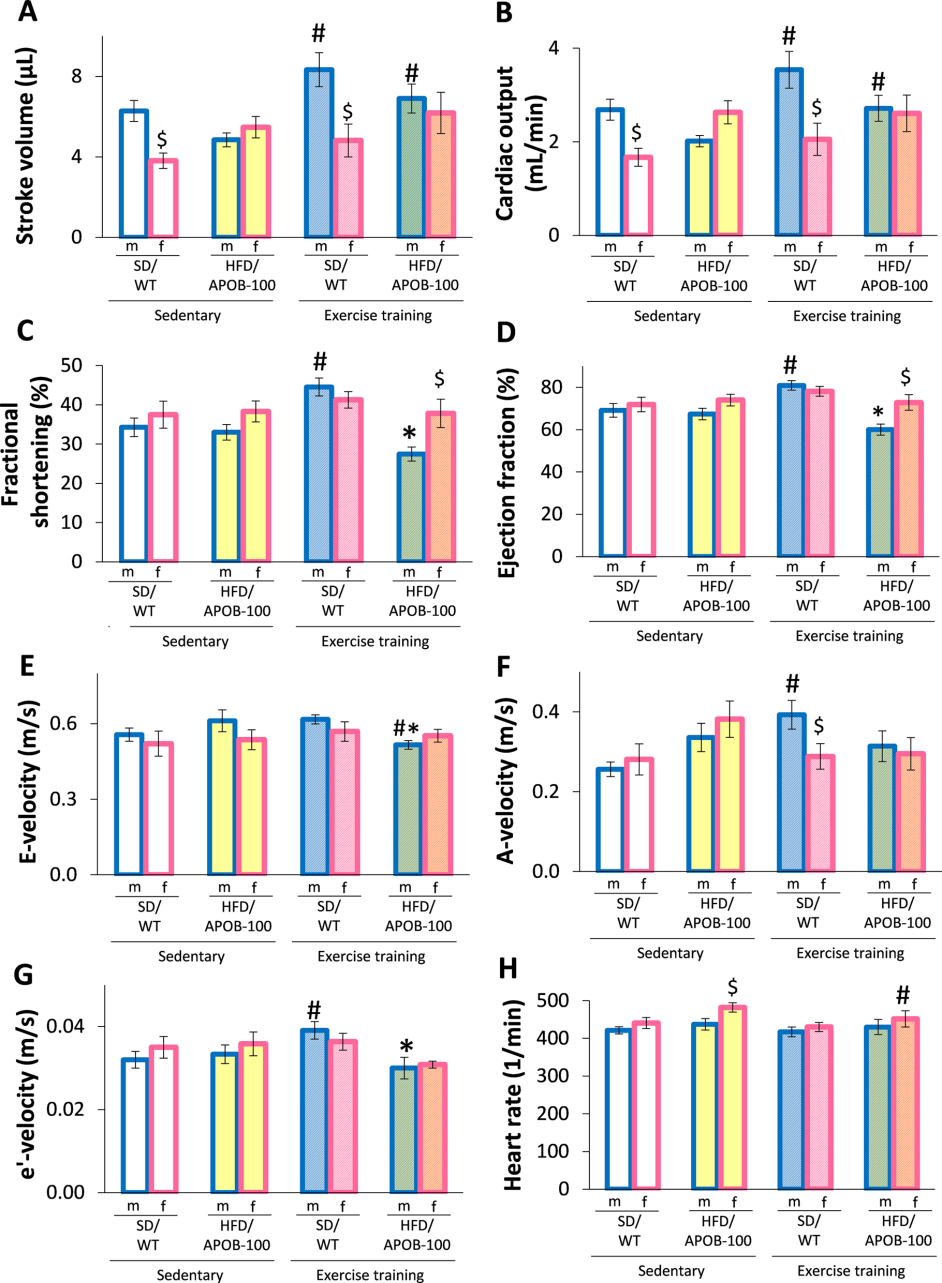
The effects of sex, MetS, and exercise training on echocardiographic parameters at 9 months of age in sedentary and exercise-trained male (m) and female (f) standard diet-fed wild-type (SD/WT) and high-fat diet-fed APOB-100 transgenic (HFD/APOB-100) mice. **A** Stroke volume, **B** Cardiac output, **C** Fractional shortening, **D** Ejection fraction, **E** E-velocity, **F** A-velocity, **G** e’-velocity, and **H** Heart rate. Values are mean ± SEM, *n* = 11–12/group, ^$^*p* < 0.05, female vs. male groups, *p < 0.05, HFD/APOB-100 vs. SD/WT groups, ^#^*p* < 0.05, ET vs. sedentary groups; Two-Way ANOVA (Holm-Sidak post hoc test) for comparisons between HFD/APOB-100 vs. SD/WT groups and ET vs. sedentary groups, and Student’s t-test or Mann–Whitney-U test for comparison between male and female groups. Kruskal–Wallis test (i.e., ANOVA on ranks) followed by Holm-Sidak post hoc test was performed in the cases of E-velocity within the male groups and fractional shortening and e’ velocity within the female groups

Transthoracic echocardiography was performed at 9 months of age to investigate whether sex, ET, or MetS could influence cardiac morphology and function (Table 2 and Fig. 4). In the sedentary SD/WT groups, females had significantly thinner inferior wall thicknesses, and smaller left ventricular end-diastolic volume, stroke volume, and cardiac output compared to males (^$^*p* < 0.05, Student’s *t* test, Table 2 and Figs. 3D, E, 4A, B). There was no significant difference in the main systolic parameters, including the fractional shortening (Fig. 4C) and ejection fraction (Fig. 4D) and the diastolic parameters, including IVRT, E-, A-, and e’-velocities and the E/A and E/e’ ratios between the sedentary SD/WT males and females (Table 2, Fig. 4E–G). There was no significant difference in the wall thicknesses and left ventricular diameters in the SD/WT males between the exercise-trained and sedentary groups (Table 2). In contrast, SD/WT females developed significantly increased diastolic inferior and systolic posterior wall thicknesses as well as significantly reduced left ventricular end-diastolic and end-systolic diameters in response to exercise, showing the echocardiographic signs of mild physiologic hypertrophy (^#^*p* < 0.05, Table 2).

#### SD/WT males developed increased left ventricular volumes, but females presented the echocardiographic signs of physiologic hypertrophy in response to exercise training

In response to ET, SD/WT males but not females developed significantly increased fractional shortening, ejection fraction, left ventricular end-systolic, and tendentiously increased end-diastolic volumes (^#^*p* < 0.05, Table 2, Fig. 4A and B). There was no statistically significant difference in the cardiac output and heart rate between the exercise-trained and sedentary SD/WT female groups (Fig. 4B and H). Exercise-trained SD/WT males had significantly increased A- and e’-velocities compared to sedentary SD/WT males, indicating an improved diastolic function (^#^*p* < 0.05, Fig. 4F and G).

#### Male sedentary HFD/APOB-100 mice developed mild HF, whereas females showed the echocardiographic signs of the beginning of LVH

In the sedentary HFD/APOB-100 groups, females had significantly increased anterior wall thicknesses (^$^*p* < 0.05, Mann–Whitney-U test) and reduced left ventricular end-systolic and end-diastolic diameters compared to males (^$^*p* < 0.05, Student’s t-test and Mann–Whitney-U test, respectively, Table 2). Notably, the diastolic anterior wall thickness was significantly increased, and the left ventricular longitudinal and cross-sectional end-diastolic and end-systolic diameters were significantly decreased in the sedentary HFD/APOB-100 females compared to the sedentary SD/WT females (*p < 0.05, Table 2). Other wall thicknesses and left ventricular end-diastolic and end-systolic volumes were not significantly different between the sedentary HFD/APOB-100 females and SD/WT females (Table 2). Notably, sedentary HFD/APOB-100 males tended to have decreased diastolic posterior, systolic, and diastolic septal wall thickness (*p* = 0.167, *p* = 0.063, and *p* = 0.099, respectively) and increased longitudinal left ventricular end-diastolic diameter (*p* = 0.107) and left ventricular end-systolic diameter (*p = 0.034) compared to sedentary SD/WT males (Table 2). Sex or MetS did not influence the main systolic parameters, including the fractional shortening (Fig. 4C) and ejection fraction (Fig. 4D) or the measured diastolic parameters, including IVRT, E-, A-, and e’-velocities (Fig. 4E–G) and the E/A ratio in the sedentary groups significantly (Table 2). Only the E/e’ was significantly reduced in the sedentary HFD/APOB-100 females compared to HFD/APOB-100 males (^$^*p* < 0.05, Student’ t-test, Table 2). Notably, the heart rate was significantly increased in the sedentary HFD/APOB-100 females compared to sedentary HFD/APOB-100 males (^$^*p* < 0.05, Student’ t-test); however, it was not significantly increased compared to sedentary SD/WT females (Fig. 4H).

#### Exercise training worsened cardiac morphology and function in HFD/APOB-100 males

Exercise-trained HFD/APOB-100 males had significantly reduced systolic anterior and inferior wall thicknesses, fractional shortening, ejection fraction, and increased cross-sectional left ventricular end-systolic diameter compared to exercise-trained SD/WT males (*p < 0.05, Table 2, Fig. 4C and D), suggesting a potentially harmful effect of ET on HFD/APOB-100 males. Indeed, exercise-trained HFD/APOB-100 males had significantly shortened IVRT and markedly reduced e’ compared to sedentary HFD/APOB-100 males (*p < 0.05, Table 2). Moreover, the left ventricular end-diastolic and end-systolic volumes, stroke volume, and cardiac output were significantly increased in the exercise-trained HFD/APOB-100 males compared to the sedentary HFD/APOB-100 males (^#^*p* < 0.05, Table 2, Fig. 4A and B).

#### Exercise training improved cardiac morphology in HFD/APOB-100 females

The exercise-trained HFD/APOB-100 females had significantly reduced diastolic posterior thickness compared to males (^$^*p* < 0.05, Student’s t-test, Table 2, Fig. 3D and E). Interestingly, the cross-sectional left ventricular end-systolic diameter was significantly decreased, and fractional shortening and ejection fraction were significantly increased in exercise-trained HFD/APOB-100 females compared to males (^$^*p* < 0.05, Student’s t-test, Table 2, Fig. 4C and D). Indeed, systolic and diastolic anterior, posterior and septal wall thicknesses were significantly reduced in the exercise-trained HFD/APOB-100 females compared to exercise-trained SD/WT females (*p < 0.05, Table 2) or sedentary HFD/APOB-100 females (^#^*p* < 0.05, Table 2). There was no significant difference in the majority of diastolic parameters, including A-, and e’-velocities, and E/A ratio between the sedentary and exercise-trained HFD/APOB-100 females (Table 2). It should be noted that the E-velocity showed a trend toward a decrease in exercise-trained HFD/APOB-100 females compared to sedentary HFD/APOB-100 females, and therefore, the E/e’ ratio was significantly higher in the exercise-trained HFD/APOB-100 females. Notably, the heart rate was significantly decreased in the exercise-trained HFD/APOB-100 females compared to the sedentary HFD/APOB-100 females (^#^*p* < 0.05, Fig. 4H).

### Exercise training resulted in cardiomyocyte hypertrophy only in females and reduced the cardiac collagen content irrespective of sex

**Fig. 5:**
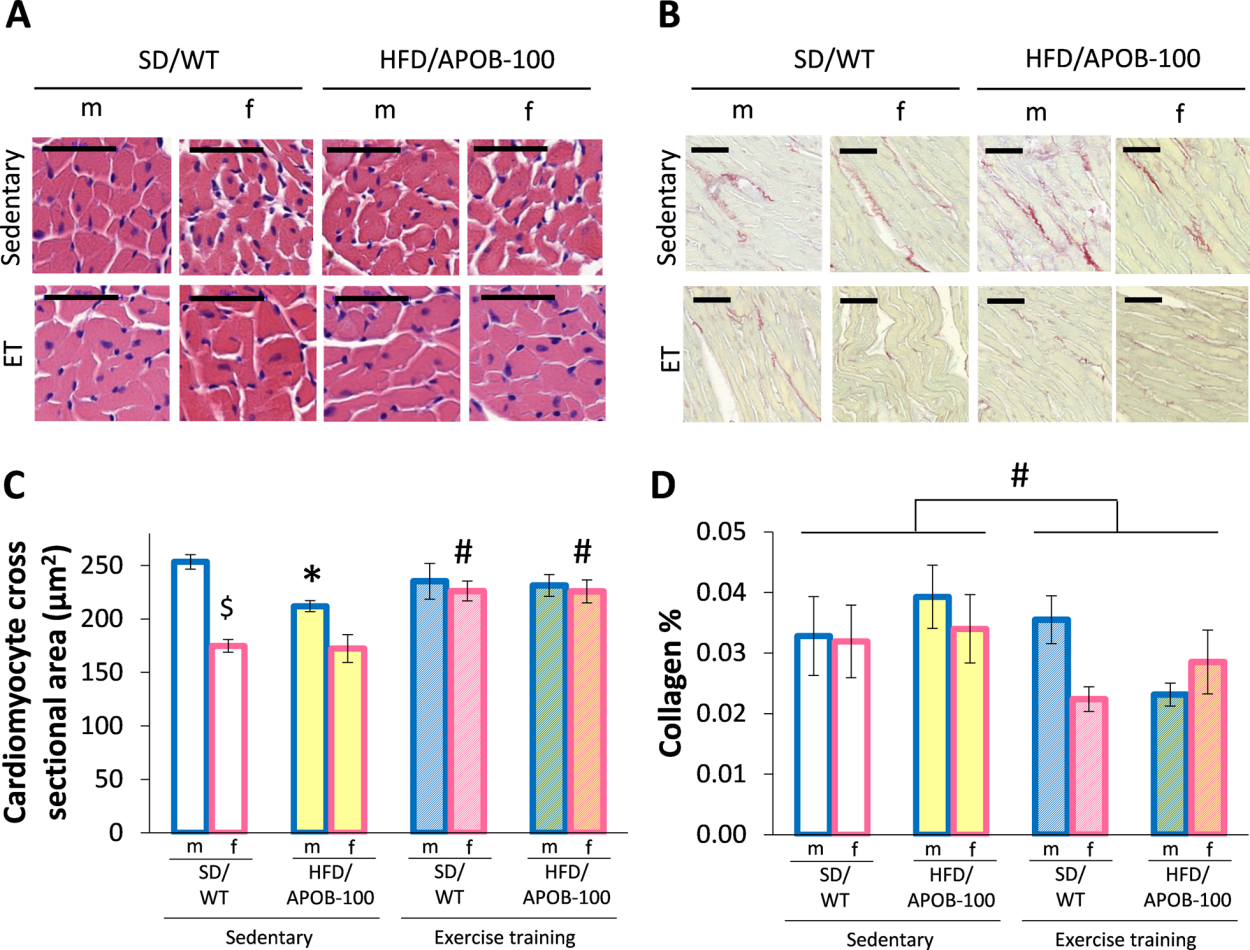
The effects of sex, MetS, and exercise training (ET) on cardiomyocyte cross-sectional area and left ventricular collagen content at 10 months of age in sedentary and exercise-trained male and female standard diet-fed wild-type (SD/WT) and high-fat diet-fed APOB-100 transgenic (HFD/APOB-100) mice. **A** Representative hematoxylin–eosin (HE, ×40 and ×100) and **B** picrosirius red/fast green-stained (PSFG, ×10) sections, **C** Cardiomyocyte cross-sectional area, and **D** Left ventricular collagen content. Values are mean ± SEM, *n* = 6/group, ^$^*p* < 0.05, female vs. male groups, *p < 0.05, HFD/APOB-100 vs. SD/WT groups, ^#^*p* < 0.05, ET vs. sedentary groups; Three-Way ANOVA (Holm-Sidak post hoc test). The black lines on the representative images mean 50 μm (Panels A and B)

To verify the echocardiographic changes, cardiomyocyte cross-sectional areas were measured on HE-stained slides (Fig. 5A), while collagen content was assessed on PSFG-stained slides (Fig. 5B). In the sedentary groups, SD/WT control females had significantly reduced (^$^*p* < 0.05, Student’s t-test, Fig. 5C), and HFD/APOB-100 females showed a tendency of decreased cardiomyocyte cross-sectional area compared to the corresponding group-matched males (Fig. 5C). In the HFD/APOB-100 groups, only sedentary males had significantly reduced cardiomyocyte cross-sectional area compared to sex-matched controls (*p < 0.05, Fig. 5C). In response to ET, cardiomyocyte cross-sectional areas were significantly increased in females (in both SD/WT and HFD/APOB-100 animals, ^#^*p* < 0.05) but were not different in males (Fig. 5C).

Interestingly, sedentary SD/WT and HFD/APOB-100 mice had similar left ventricular collagen content in both sexes (Fig. 5D). However, exercise-trained SD/WT and HFD/APOB-100 animals had significantly reduced left ventricular collagen content (^#^*p* < 0.05, Fig. 5D). Notably, in the exercise-trained groups, SD/WT females had reduced left ventricular collagen content compared to SD/WT males (Fig. 5D). It should also be mentioned that exercise-trained male HFD/APOB-100 mice tend to show less left ventricular collagen content compared to sedentary male HFD/APOB-100 mice (*p* = 0.15, Fig. 5D).

### The effects of sex, MetS- or ET on the left ventricular gene expression changes

#### Females showed a higher expression level of the left ventricular leptin receptor gene compared to males

**Fig. 6:**
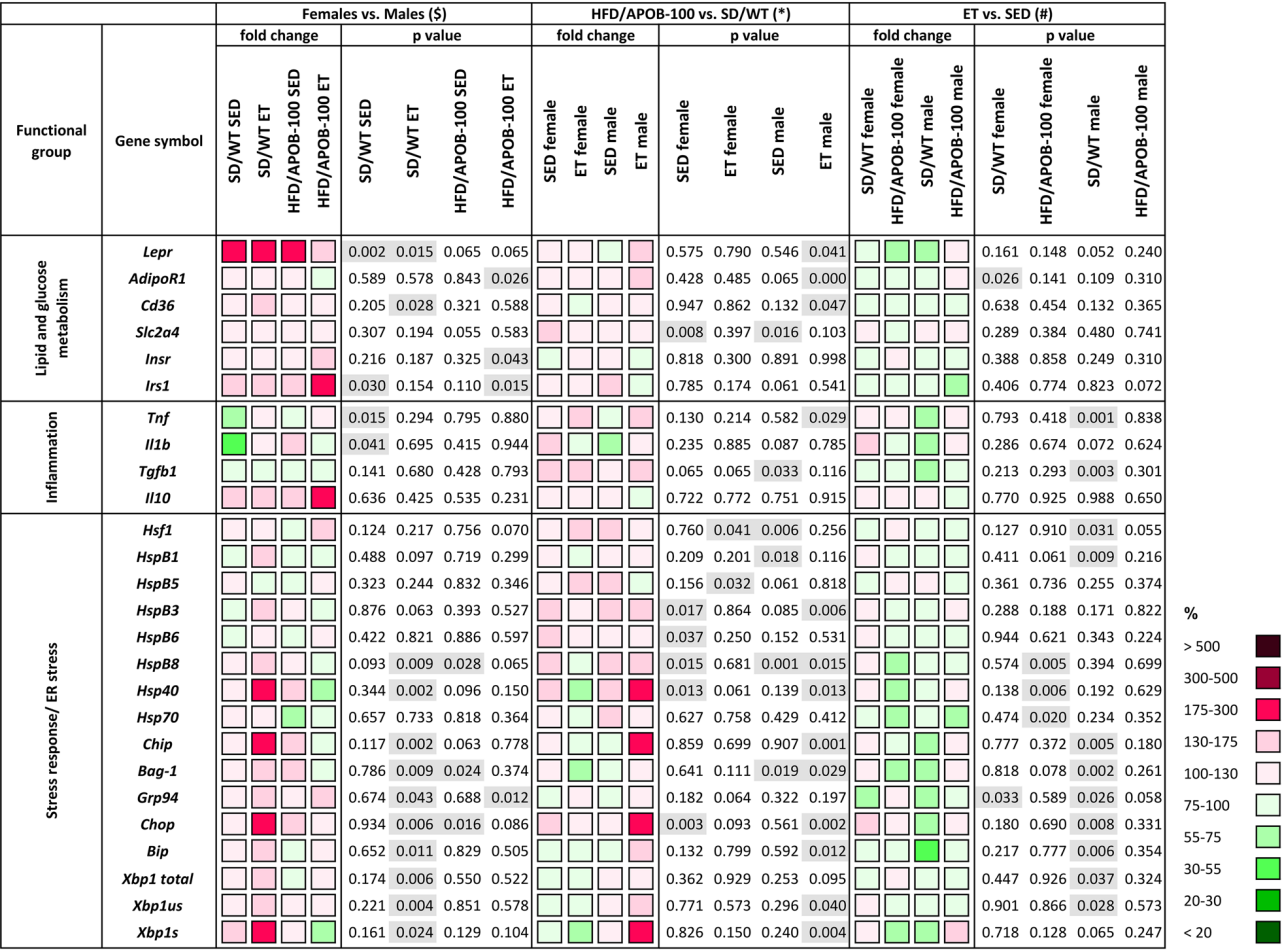
The effects of sex, Mets and exercise training (ET) on left ventricular gene expression at 10 months of age in sedentary and exercise-trained male and female standard diet-fed wild-type (SD/WT) and high-fat diet-fed APOB-100 transgenic (HFD/APOB-100) mice (*n* = 6 animals/group). For the female vs. male comparison, the relative expression of target genes in females was compared to the expression levels detected in males (results are given in percentage, where the male groups = 100%). For HFD/APOB vs. SD/WT comparisons, the relative expression of target genes in high-fat diet-fed APOB-100 transgenic animals was compared to the expression levels detected in standard diet-fed wild-type animals (results are given in percentage, where SD/WT groups = 100%). For the ET vs. SED comparison, the relative expression of target genes in exercise-trained animals was compared with the expression levels detected in the corresponding sedentary groups (results are given as a percentage, where the corresponding value for the sedentary groups = 100%)

We also investigated whether the sex, MetS- or ET-associated left ventricular alterations are accompanied by changes in gene expression (Fig. 6). Therefore, we isolated total RNA from left ventricle tissue samples, and expression levels of genes involved in the regulation of glucose and lipid metabolism, inflammation, and stress response were measured by qPCR (for details, see Table 1).

Sex-based differences were analyzed by comparing the values of female vs. male groups, where the values for the corresponding male animals were considered as 100%. The left ventricular expression level of the leptin receptor encoding gene, *Lepr,* was significantly higher in female animals compared to corresponding male animals in the sedentary and exercise-trained SD/WT groups (177%, ^$^*p* < 0.01, Student’s t-test; 219%, ^$^*p* < 0.05, Mann–Whitney U-test, respectively) (Fig. 6). Moreover, the insulin receptor substrate 1 (*Irs1)* gene, encoding a central regulator of insulin signaling, showed significantly higher left ventricular expression in the exercise-trained HFD/APOB-100 females than in males (202%, ^$^*p* < 0.05, Student’s t-test). Interestingly, left ventricular mRNA levels of several genes, involved in the regulation of stress response (*Hsp40*: 182%, ^$^*p* < 0.01, Student’s t-test; *Chip*: 202%, ^$^*p* < 0.01, Mann–Whitney U-test) and ER stress (*Chop*: 198%, ^$^*p* < 0.01, Student’s t-test; *Xbp1s*: 207%, ^$^*p* < 0.05, Student’s t-test), were higher in the left ventricles of exercise-trained SD/WT females compared to exercise-trained SD/WT males (Fig. 6).

#### Exercise-trained HFD/APOB-100 males showed left ventricular overexpression of stress response-related genes

The effects of MetS were measured by comparing the values of the HFD/APOB-100 groups to the SD/WT controls by qPCR (for all comparisons, values in wild-type mice are 100%) (Fig. 6). Although many of the studied genes showed non-significantly higher expression levels in the HFD/APOB-100 animals, only *Hsp40*, *Chip, Chop*, and *Xbp1s* were remarkably increased (177%, *p < 0.05, Student’s t-test; 179%, *p < 0.01, Student’s t-test; 188%, *p < 0.01, Student’s t-test; 187%, *p < 0.01, Mann–Whitney U-test) in the exercise-trained male HFD/APOB-100 group compared to the exercise-trained SD/WT male group (Fig. 6). Comparing the exercise-trained and sedentary groups (where the values of the corresponding sedentary groups were considered as 100%), most of the investigated genes tended to be slightly repressed in the exercise-trained animals. Among them, *Bip1* showed the most remarkable decrease in response to ET in the SD/WT male animals (53%, ^#^*p* < 0.01, Student’s t-test) (Fig. 6).

## Discussion

In the present study, we showed the first time that 10-month-old male and female HFD/APOB-100 mice have different cardiovascular responses and cardiac gene expressional changes to MetS and ET.

In our present study, there was no significant sex-based difference in the serum LDL/HDL ratio and the HOMA-IR values in the sedentary SD/WT groups. However, the sex-based differences in the heart size are clearly seen in our present study in the heart weight, cardiomyocyte cross-sectional area, stroke volume, and cardiac output in the wild-type sedentary females compared to the wild-type sedentary males. These findings are in accordance with the results of both the Framingham Heart Study and Multi-Ethnic Study of Atherosclerosis. The aforementioned clinical studies demonstrated that left ventricular volume and mass are significantly lower in healthy women than men, even after adjusting for height and body surface area [33, 34].

In our present study, both sexes in the HF/APOB-100 groups had higher serum LDL- and HDL-cholesterol levels compared to the sex-matched SD/WT groups, indicating the development of hypercholesterolemia in our MetS model. However, HFD/APOB-100 females presented lower serum LDL- and HDL-cholesterol levels than males. In addition, only HFD/APOB-100 males developed insulin resistance that was characterized by significantly higher HOMA-IR due to their markedly elevated fasting blood glucose levels than sex-matched SD/WT mice [8]. Thus, our present findings on the sex-based differences in insulin resistance and serum lipoprotein levels are in accordance with the fact that premenopausal women have lower cardiovascular risk than age-matched men [35, 36]. The echocardiographic and histologic findings in our mouse model of MetS are in line with the clinical observations that women develop more frequently LVH and HFpEF, whereas men more commonly present HFrEF with dilated ventricles and thinner walls [7, 16, 18]. Indeed, in our present study, HFD/APOB-100 females started to develop a mild LVH characterized by increased anterior wall thicknesses and reduced left ventricular diameters at 10 months of age. Despite the echocardiographic signs of a mild LVH, the heart weight and cardiomyocyte cross-sectional area remained smaller in sedentary HFD/APOB-100 females than males, probably, due to the smaller body and heart size of females. In contrast, HFD/APOB-100 males presented slightly dilated ventricles, and thinner left ventricular walls with reduced cardiomyocyte cross-sectional area, suggesting the beginning of the HFrEF development. Notably, ejection fraction was preserved in both sexes, and there was no significant difference in the left ventricular collagen content between the sedentary wild-type and HFD/APOB-100 groups, excluding the presence of a severe maladaptive remodeling in the HFD/APOB-100 mice. The pathologic cardiac remodeling would probably be more advanced at a later follow-up time in the HFD/APOB-100 mice.

It is well known that regular ET can reduce cardiovascular risk [21]. In our present study, ET reduced the body weight in HFD/APOB-100 mice, suggesting a beneficial effect of ET in both sexes. In contrast, serum LDL- and HDL-cholesterol levels and insulin resistance failed to improve in response to ET in both sexes in the HFD/APOB-100 mice, probably due to the solid genetic background of MetS in APOB-100 mice. In response to ET, physiological hypertrophy could be associated with a mild increase in cardiac mass with preserved or increased contractile function and individual cardiomyocyte growth in both length and width without fibrosis [37]. In our present study, the heart weight failed to increase in response to ET in the SD/WT females compared to the sedentary SD/WT females. However, exercise-trained SD/WT females started to develop a phenotype similar to that of physiologic hypertrophy, where ejection fraction was unchanged, and probably, the cardiomyocyte width was more increased according to the cardiomyocyte cross-sectional areas and increased inferior wall thicknesses measured in the cross-sectional echocardiographic view. In contrast, exercise-trained SD/WT males had no significantly higher end-systolic and end-diastolic volumes and no change in cross-sectional areas, suggesting a higher grade growth in cardiomyocytes length than widths leading to increased ejection fraction, stroke volume, and cardiac output. Thus, cardiac compensation in response to ET seems to be different in SD/WT males than in females.

Several guidelines for HF treatment also recommend ET beyond pharmacologic strategies; however, only limited data is available on the sex-based differences in response to ET in HF patients [19, 23, 24]. In our present study, ET seems to be harmful in HFD/APOB-100 males worsening HF by further dilating the left ventricles and reducing the ejection fraction. However, exercise-trained HFD/APOB-100 females presented reduced wall thicknesses with preserved ejection fraction by echocardiography compared to sedentary HFD/APOB-100 females. In response to ET, HFD/APOB-100 females seem to respond by reducing the pathologic hypertrophy showed in the sedentary HFD/APOB-100 females. However, as demonstrated by histology, cardiomyocyte growth is also present in exercise-trained HFD/APOB-100 females, similarly to the exercise-trained SD/WT females. In contrast, we showed increased left ventricular volumes as a physiologic response to ET in SD/WT males and a pathophysiologic response to MetS in HFD/APOB-100 males. Therefore, in our present study, the two stressors (i.e., MetS and ET) seem to result in the exhaustion of the compensatory mechanisms leading to decreased ejection fraction and worsening HF in the HFD/APOB-100 males. Our present finding seems to be in accordance with the results of HF-ACTION trial reporting that women suffering from HF had lower all-cause mortality or all-cause hospitalization in response to regular ET [38]. It is important to note that the optimal intensity and type of exercise always depend on the current physical fitness and health condition of the patient. Therefore, personalization is essential when it is used for the treatment of cardiovascular abnormalities [39, 40].

The left ventricular expression of several genes associated with glucose and lipid metabolism, inflammation, and stress response were investigated by qPCR to investigate the underlying mechanisms of the morphologic and functional results. The significant overexpression of the leptin receptor mRNA level in both the sedentary and exercise-trained SD/WT females was the most remarkable sex-dependent difference. Although the serum leptin concentrations of male and female animals were found to be similar in our previous study, an improved leptin sensitivity may contribute to the lower susceptibility of the female mice to diet-induced metabolic disturbances [8]. In the heart, leptin may influence the structure and function of cardiomyocytes and could regulate inflammation and glucose and fatty acid metabolism [41–43]. In vitro and in vivo experiments suggest that leptin treatment may induce cardiomyocyte hypertrophy [42, 44], while others found antihypertrophic effects of leptin [45]. Moreover, in our present study, the docking protein insulin receptor substrate-1 (*Irs1)* was significantly overexpressed in exercise-trained HFD/APOB-100 female hearts compared to male hearts. Interestingly, the abnormal function of IRS1 protein is involved in the development of insulin resistance [46], and the loss of IRS proteins is suggested to be a link between diabetes and cardiac insulin resistance in HF. Indeed, heart-specific lack of these genes could lead to reduced ventricular mass and cardiac failure [47, 48]. Members of the different HSP families cooperate with each other and their co-chaperones, forming a complex network to maintain the normal protein homeostasis in different tissues under stress conditions [49–51]. Increased HSP expression by ET was proved to be protective against ischemia/reperfusion injury [52]. In diabetes mellitus, reduced HSP expression is associated with the decreased ability of insulin-sensitive tissues to respond to stress [53]. Previously, we have also demonstrated that hyperlipidemia attenuated heat shock response in rat hearts [54]. HSP40 and CHIP are co-chaperones of HSP70, regulating ATP hydrolysis/substrate binding of HSP70 and the proteasomal degradation of misfolded proteins, respectively, while CHOP and XBP1 are ER stress-related proteins [51]. Accumulation of unfolded proteins can lead to the alternative splicing of the mRNA of XBP1, a transcription factor that is an important regulator of ER stress [55, 56]. We found here that the mRNA levels of *Hsp40*, *Chip*, *Chop*, and *Xbp1s* in sedentary SD/WT females were equal to or slightly higher than that of sedentary SD/WT males. In response to regular training, these factors were slightly increased in the females while decreased in males, which led to a remarkable difference between exercise-trained SD/WT males and females. Cardiac hypertrophy was described to be accompanied by the accumulation of misfolded proteins [57]. Therefore, the increased expression of stress factors could be a compensatory mechanism in the heart of the female mice subjected to regular ET in our present study. Moreover, the same factors also showed a slight increase in hyperlipidemic HFD/APOB-100 males due to ET compared to SD/WT males. Therefore, trained HFD/APOB-100 males showed significantly higher expression levels than trained SD/WT males. This suggests that ET or hyperlipidemia alone did not influence HSP expression, whereas the combination of the two effects was able to induce stress response in the hearts of male mice.

## Limitations

Our study is not without limitations. Our results regarding altered cardiac gene expression due to sex, MetS, and ET are based on expressional changes of selected genes; however, confirmation of these gene expression changes at the protein level and direct measurement of the full transcriptome and proteome should be performed in the future. Moreover, additional studies providing more in-depth mechanistic insight and more functional assessment, including left ventricular pressure–volume changes and blood pressure measurement, should be carried out. Although our study does not specify which cell type (i.e., cardiomyocyte, endothelial cell, fibroblast, smooth muscle cell, etc.) may be responsible for the observed alterations of cardiac gene expression due to sex, MetS, or ET, the contribution of cardiomyocytes is likely the most significant [58].

## Perspectives and significance

In conclusion, both HFD/APOB-100 males and females developed obesity and hypercholesterolemia; however, only males presented insulin resistance. ET did not change these metabolic parameters significantly. HFD/APOB-100 males showed echocardiographic signs of mild HF with thinner walls and dilated ventricles, whereas females developed a starting LVH assessed by echocardiography and histology. In response to ET, SD/WT males developed increased left ventricular volumes, and females presented physiologic hypertrophy. In contrast, exercise-trained HFD/APOB-100 males presented worsening HF with reduced ejection fraction. On the contrary, exercise-trained HFD/APOB-100 females reversed the echocardiographic signs of LVH. We conclude that sex, MetS, and ET alters the gene expression pattern of the myocardium which may be involved in the development of sex-specific cardiac alterations in the state of MetS or to ET. Based on our present exploratory results, future studies should be carried out to investigate the precise role of specific genes, particularly *Lepr*, *Irs1*, and stress-related genes, in the development of sex-specific cardiac responses to MetS and ET to obtain deeper mechanistic insights. There is also an urgent need for clinical trials to investigate the effects of ET on the development of metabolic syndrome-associated heart failure in both sexes.

## Data Availability

The data sets used and/or analyzed during the current study are available from the corresponding author on reasonable request.

## Abbreviations

APOB-100: Apolipoprotein B-100
A-velocity: Atrial flow velocity
CVDs: Cardiovascular diseases
ER: Endoplasmic reticulum
ET: Exercise training
E-velocity: Early flow velocity
e’-velocity: Septal mitral annular velocity
FS: Fractional shortening
HDL: High-density lipoprotein
HE: Hematoxylin–eosin
HF: Heart failure
HFD: High-fat diet
HFpEF: Heart failure with preserved ejection fraction
HFrEF: Heart failure with reduced ejection fraction
HSP: Heat shock protein
HOMA-IR: Homeostatic model assessment for insulin resistance
HSL: Hue-Saturation-Luminance
IVRT: Isovolumic relaxation time
LDL: Low-density lipoprotein
LVEDD: Left ventricular end-diastolic diameter
LVEDV: Left ventricular end-diastolic volume
LVESD: Left ventricular end-systolic diameter
LVESV: Left ventricular end-systolic volume
LVH: Left ventricular hypertrophy
MetS: Metabolic syndrome
PBS: Phosphate-buffered saline
PSFG: Picrosirius red and fast green
RGB: Red–Green–Blue
SD: Standard diet
T2DM: Type 2 diabetes mellitus
qPCR: Quantitative real time polymerase chain reaction
VLDL: Very low-density lipoprotein
WT: Wild type
